# Right S3 segmentectomy for lung cancer with partial anomalous pulmonary venous return in the right upper pulmonary vein: A case report

**DOI:** 10.1111/1759-7714.15266

**Published:** 2024-02-23

**Authors:** Tetsuya Isaka, Takuya Nagashima, Kotaro Murakami, Shunsuke Shigefuku, Noritake Kikunishi, Naoko Shigeta, Hiroyuki Ito

**Affiliations:** ^1^ Department of Thoracic Surgery Kanagawa Cancer Center Yokohama Japan

**Keywords:** emphysema, partial anomalous pulmonary venous return, segmentectomy

## Abstract

Partial anomalous pulmonary venous return (PAPVR) is a rare congenital malformation where the pulmonary vein partially refluxes into the venous system. Here, we present the first robotic‐assisted right S3 segmentectomy in a 70‐year‐old male with early‐stage lung cancer and PAPVR in the right upper pulmonary vein. The patient, with suspected primary lung cancer (11 mm diameter, pure solid appearance in right S3 segment), exhibited clinical stage T1bN0M0 stage IA2. Preoperative computed tomography revealed severe lung emphysema, and right V1–3 returned directly to the superior vena cava. However, no signs of right‐sided heart failure were observed, and echocardiogram was normal with a pulmonary‐to‐systemic blood flow ratio of 1.4. Successful robot‐assisted right S3 segmentectomy with hilar nodal dissection was performed, and the patient was discharged on the sixth postoperative day without complications. One year postoperatively, there has been no recurrence of lung cancer or respiratory/right‐sided heart failure symptoms.

## Graphical abstract

We report a rare case of early‐stage lung cancer along with partial anomalous pulmonary venous return with a small left‐to‐right shunt. Robot‐assisted right S3 segmentectomy was performed safely without postoperative cardiac and respiratory complications in low respiratory patients with partial anomalous pulmonary venous return in the same lobe.

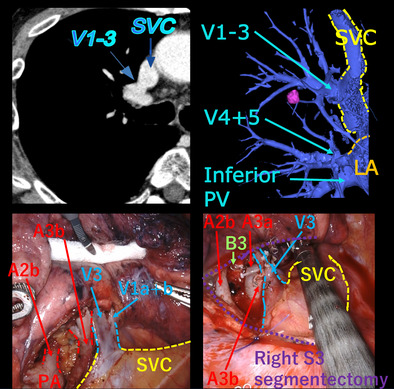

## INTRODUCTION

Partial anomalous pulmonary venous return (PAPVR) is a congenital malformation where a portion of the pulmonary vein returns abnormally into the venous system. Its incidence has been reported to be approximately 0.1% in the adult population based on computed tomography (CT).^1^, ^2^ Patients are typically asymptomatic and it is incidentally detected during the investigation of other diseases.^3^ However, symptoms of right‐sided heart failure, such as dyspnea and palpitations, may arise as the right and left shunts enlarge.^3^ A pulmonary‐to‐systemic blood flow ratio (Qp/Qs) of 1.5–2.0 or higher is considered an indication for revascularization.^4^ Here, we report the first case of robot‐assisted right S3 segmentectomy for a patient with early lung cancer with PAPVR in the right upper pulmonary vein.

## CASE REPORT

**FIGURE 1:**
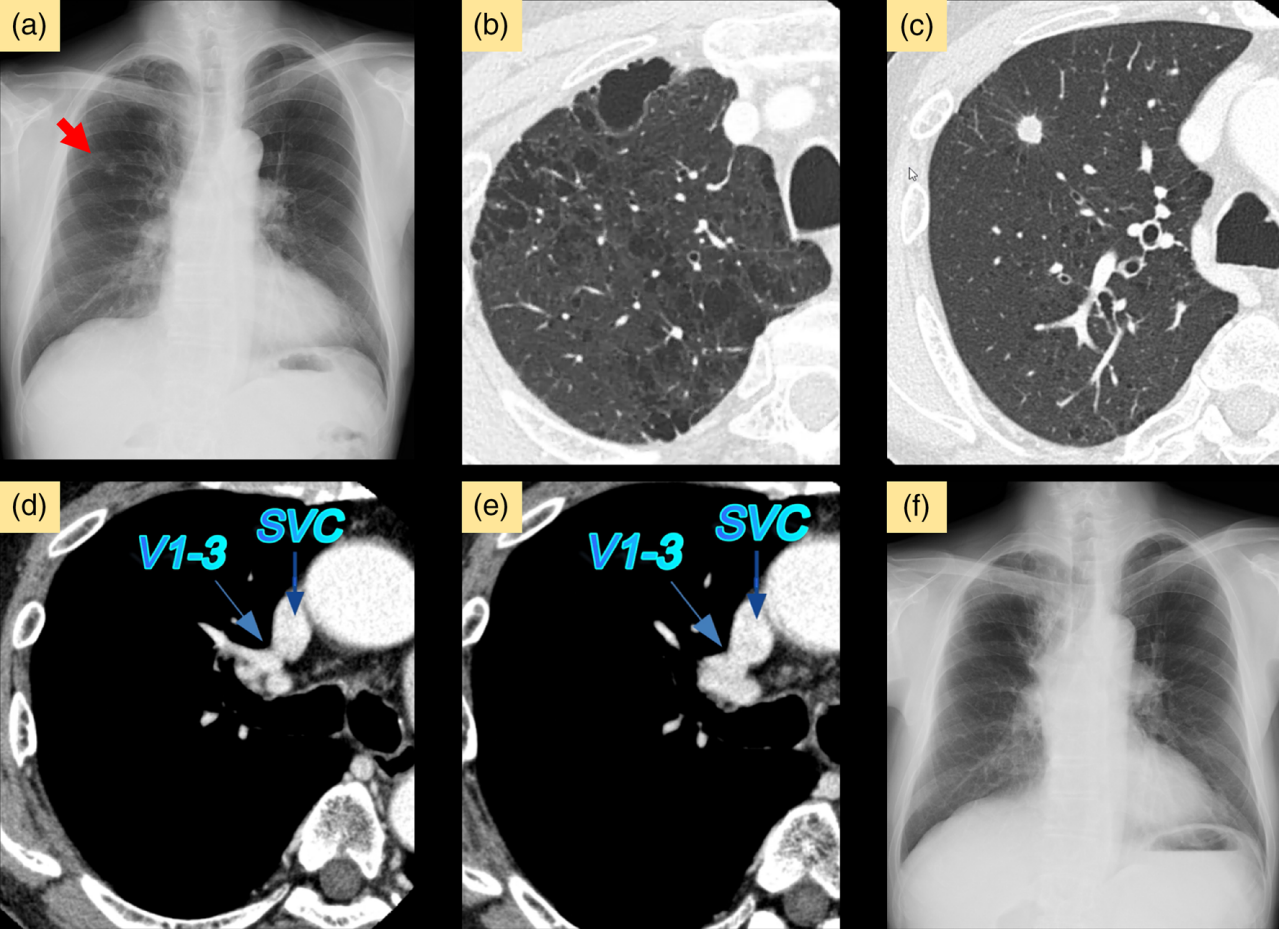
(a) Chest x‐ray revealed a nodule in the right upper lung field (red arrow) without cardiac enlargement. (b) Contrast‐enhanced computed tomography revealed severe emphysematous lung changes with diffuse low attenuation areas in the lung fields. (c) A pure solid tumor with 11 mm diameter in the S3 of the right upper lobe. (d, e) V1–3 refluxed through the right upper lobe and returned directly into the superior vena cava. (f) After 4 months following surgery, a chest x‐ray revealed that the remaining right upper lobe was well‐inflated, and no cardiac enlargement was observed. SVC, superior vena cava.

**FIGURE 2:**
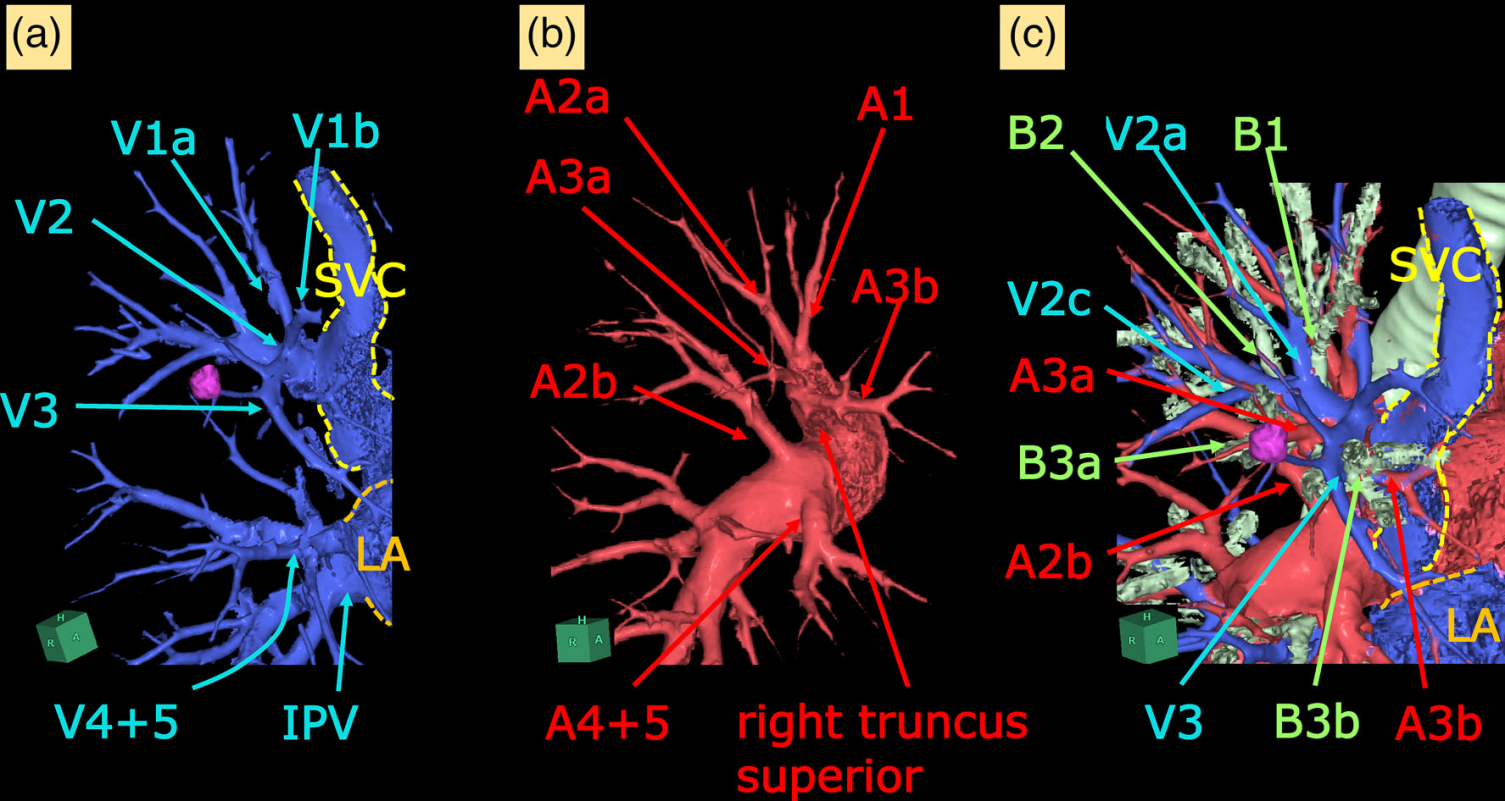
(a) Three‐dimensional images of the bronchial and vascular structures of the patient revealed that an abnormal V1–3 returning into the superior vena cava consisted of V1a, V1b, V2, and V3. (b) The bifurcation of pulmonary arteries was normal; the right main pulmonary artery branched into the right truncus superior and A2a, whereas the truncus superior pulmonary artery branched into A1, A2a, A3a, and A3b. (c) The right upper bronchus branched into B1, B2, and B3 as per normal branching. The A3a, A3b, V3, and B3 were dissected during S3 segmentectomy, located caudal to V2. LA, left atrium; SVC, superior vena cava.

**FIGURE 3:**
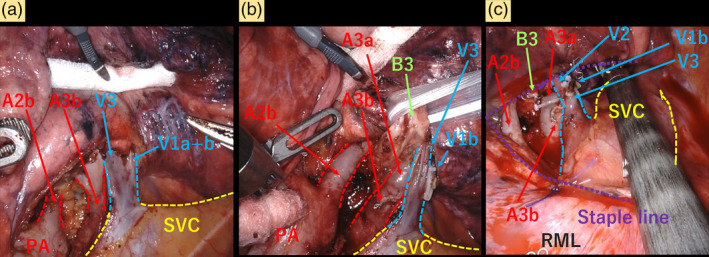
(a) Intraoperative findings showing that an abnormal V1–3 returned directly into the superior vena cava. The caudal side of abnormal V1–3 demonstrated A3b branching from the right truncus superior. (b) A3a was identified just behind the A3b after A3b was divided with a stapler. B3 was located on the caudal side of abnormal V1–3 within the space between A3 and A2b. (c) V2 was located just behind V3 and V1b after V3 and V1b were clipped and divided. After dividing V1b, V3, A3a, A3b, and B3, the lung parenchyma of the S3 segment was dissected using a stapler. RML, right middle lobe; SVC, superior vena cava.

A 70‐year‐old male presented with suspected primary lung cancer (clinical stage T1bN0M0, stage IA2) based on a chest radiograph during physical examination. He was undergoing inhalation therapy for emphysema. Chest x‐ray revealed a nodule in the right upper lung field, without cardiac enlargement (Figure 1a). Chest CT revealed severe lung emphysema and an 11 mm pure solid tumor in the right S3 segment (Figure 1b,c). Preoperative contrast CT revealed abnormal return of the right V1–3 directly into the superior vena cava (SVC), with V4 + 5 and the inferior pulmonary vein returning normally to the left atrium (Figures 1d,e and 2a,c). The bronchial and pulmonary artery branches of the right upper lobe were normal (Figure 2b,c). His preoperative forced expiratory volume in 1 s/forced vital capacity ratio was 58.7%. There were no signs of heart failure, and echocardiogram was normal, with a pulmonary‐to‐systemic blood flow ratio (Qp/Qs) of 1.4 and a tricuspid regurgitation pressure gradient of 35 mmHg, without an atrial septal defect. Robot‐assisted right S3 segmentectomy was performed with five ports and CO_2_ insufflation pressure under 8 mmHg (Figure 3a–c). The operation took 149 min with a blood loss of 3 mL. The chest drain was removed on postoperative day 3, and the patient was discharged on postoperative day 6 without any complications. Postoperative chest x‐ray revealed that the remaining right upper lobe was well‐inflated, and no enlargement of the cardiac shadow was observed (Figure 1f). The pathological findings indicated invasive papillary adenocarcinoma (pT1bN0M0, stage IA2) with a 15‐mm margin. One year following surgery, no lung cancer recurrence or any respiratory or right‐sided heart failure symptoms were observed with a Qp/Qs of 1.3 in the echocardiogram.

## DISCUSSION

Surgical strategies for lung cancer in patients with PAPVR depend on factors, such as the side of PAPVR, lung cancer location, left–right shunt magnitude, and extent of lung resection.^3^, ^4^, ^5^ If lung cancer is contralateral to PAPVR, there is a potential risk of intraoperative ventilation‐perfusion mismatch and pulmonary artery pressure elevation during lung cancer surgery with isolated lung ventilation. Consequently, revascularization before lung cancer surgery becomes necessary.^3^, ^5^, ^6^ If PAPVR is present in another lung lobe on the same side as the resected lung, intraoperative revascularization should be considered for patients at high risk of right‐sided heart failure due to volume and pressure overload via left–right shunt.^3^, ^4^, ^5^ In patients with PAPVR in the resected lung, the risk of postoperative right heart failure is considered low because the right and left shunts improve simultaneously with resection.^3^, ^4^, ^5^ In reports of 15 cases, including the present case, PAPVR was diagnosed preoperatively in seven cases, whereas it was identified intraoperatively in the other cases (Table 1).^7^, ^8^, ^9^, ^10^, ^11^, ^12^, ^13^, ^14^, ^15^, ^16^, ^17^, ^18^ Right upper lobectomy was the most common procedure (nine cases), and these cases had PAPVR with right V1–3 returning to the SVC or azygos vein.

**TABLE 1:** Previous and present cases of lung cancers with partial anomalous pulmonary venous return (PAPVR) at the same resected lung lobe.

Year, authors	Age/sex	Symptom	Diagnosis of PAPVR	Tumor size (cm)	Clinical stage, histology	Procedure	Approach	Anomalous PV	Drained vein	Qp/Qs	Echocardiography	Postoperative complication
1995, Takamori et al.^7^	68/M	None	Intraop	Unknown	T3N0M0, SQ	LUL	Thoracotomy	Lt. V1–5	Lt. BCV	Unknown	Normal	Unknown
2004, Miwa et al.^8^	79/F	Unknown	Intraop	Unknown	Unknown, carcinoid	LUL	Thoracotomy	Lt. V1–5	Lt. BCV	Unknown	Normal	None
2006, Sasaki et al.^9^	59/M	Unknown	Intraop	Unknown	T2N0M0, SQ	RUL	Unknown	Rt. V1–3	SVC	Unknown	High RVDd, 34 mm	Unknown
2006, Galetta et al.^10^	62/M	Unknown	Intraop	Unknown	T2N0M0, NSCLC	RUL	Unknown	Rt. V1–3	SVC/azygos	Unknown	Normal	None
2008, Tanaka et al.^11^	81/M	None	Intraop	Unknown	T1N0M0, AD	RUL	Minithoracotomy	Rt. V1–3	SVC	Unknown	Normal	None
2009, Pryshchepau et al.^12^	80/M	Unknown	Intraop	Unknown	T2N0M0, carcinoma	RUL	VATS	Rt. V1–3	SVC	Unknown.	Normal	Unknown
2014, Asakura et al.^13^	64/M	Unknown	Preop	2.0	T1aN0M0, AD	LUL	Thoracotomy	Lt. V1–5	Lt. BCV	2.0 cardiac catheterization	Mod MR, high LVDd, and EF 53%	None
2015, Liu et al.^14^	48/M	Cough and blood sputum	Preop	Unknown	Unknown, SQ	RPN	Thoracotomy	Rt. PV	IVC	Unknown	Normal	None
2015, Heineman et al.^15^	61/F	Unknown	Intraop	Unknown	T1bN0M0, unknown	RUL	Thoracotomy	Rt. V1–3	Azygos	Unknown	Normal	None
2016, Inafuku et al.^16^	59/M	None	Preop	4.5	T2aN0M0, AD	RUL	VATS	Rt. V1–3 (excluding V2t)	SVC	Unknown	Normal	None
2017, Kawasaki et al.^3^	45/M	None	Preop	2.5	T1bN0M0, AD	RUL	VATS	Rt. V1–3	Azygos	1.08	Normal	None
2019, Singhal et al.^4^	70/M	None	Intraop	1.9	Unknown	RUL	VATS	Rt. V1–3	SVC	Unknown	Unknown	None
2020, Verma et al.^17^	73/M	None	Preop	2.0	T1bN0M0, AD	LUL	Thoracotomy	Lt. V1–3	Lt. BCV	Unknown	Unknown	None
2022, Su et al.^18^	60/M	None	Preop	1.0	Unknown	RUL	VATS	Rt. V1 + V3ac + V2b	SVC	Unknown	Normal	None
2023, Isaka et al. (present case)	70/M	None	Preop	1.1	T1bN0M0, AD	Rt. S3 SEG	RATS	Rt. V1–3	SVC	1.4	Normal	None

Abbreviations: AD, adenocarcinoma; BCV, brachiocephalic vein; EF, ejection fraction; IVC, inferior vena cava; Lt., left; LUL, left upper lobectomy; LVDd, left ventricular diastolic dimension; Mod MR, moderate mitral regurgitation; NSCLC, non‐small cell lung cancer; PAPVR, partial anomalous pulmonary venous return; PV, pulmonary vein; RATS, robot‐assisted thoracic surgery; RPN, right pneumonectomy; Rt., right; RUL, right upper lobectomy; RVDd, right ventricular diastolic dimension, SEG, segmentectomy; op, operative; SQ, squamous cell carcinoma; SVC, superior vena cava; VATS, video‐assisted thoracic surgery.

This case showed no symptoms of right heart failure, and echocardiographic findings were normal, suggesting a small left–right shunt volume. Most patients had no symptoms associated with PAPVR, and echocardiography of 11 of 13 patients was normal (Table 1). In patients with low shunt volume and low risk of right heart failure, a limited resection, including segmentectomy may preserve respiratory function and is considered a less invasive technique than lobectomy, especially in patients with low pulmonary function. If the shunt volume is large, lobectomy is necessary to reduce the right cardiac load. Given the patient's low pulmonary function due to emphysema and the early‐stage nature of the lung cancer, segmentectomy was chosen over right upper lobectomy, prioritizing the preservation of pulmonary function over shunt improvement.

Thoracotomy is a common approach for lung cancer resection in patients with PAPVR on the same lobe (Table 1). Robot‐assisted thoracic surgery is a feasible approach in segmentectomy requiring vascular and bronchial processing in the restricted space because the surgical field is extended under CO_2_ insufflation, and multiple articulations of robotic arms enable thoracic surgeons to achieve fine manipulation.

This case report presents a rare case of early‐stage lung cancer along with PAPVR with a small left‐to‐right shunt. Robot‐assisted right S3 segmentectomy was performed safely without postoperative cardiac and respiratory complications in low respiratory patients with PAPVR in the same lobe.

## AUTHOR CONTRIBUTIONS

All authors had full access to the data in this study and take responsibility for the integrity of the data and the accuracy of the data analysis. Conceptualization: Tetsuya Isaka and Hiroyuki Ito. Investigation: Tetsuya Isaka and Hiroyuki Ito. Resources: Tetsuya Isaka, Takuya Nagashima, Kotaro Murakami, Shunsuke Shigefuku, Noritake Kikunishi, Naoko Shigeta and Hiroyuki Ito. Writing–original draft: Tetsuya Isaka. Writing–review and editing: Takuya Nagashima, Kotaro Murakami, Shunsuke Shigefuku, Noritake Kikunishi, Naoko Shigeta and Hiroyuki Ito. Supervision: Hiroyuki Ito.

## CONFLICT OF INTEREST STATEMENT

All authors declare no conflict of interest.
